# REACTIN: Regulatory activity inference of transcription factors underlying human diseases with application to breast cancer

**DOI:** 10.1186/1471-2164-14-504

**Published:** 2013-07-26

**Authors:** Mingzhu Zhu, Chun-Chi Liu, Chao Cheng

**Affiliations:** 1 Department of Genetics, Geisel School of Medicine at Dartmouth, Hanover, New Hampshire 03755, USA; 2 Institute of Genomics and Bioinformatics, National Chung Hsing University, Taichung, Taiwan; 3 Agricultural Biotechnology Center, National Chung Hsing University, Taichung, Taiwan; 4 Institute for Quantitative Biomedical Sciences, Geisel School of Medicine at Dartmouth, Lebanon, New Hampshire 03766, USA; 5 Norris Cotton Cancer Center, Geisel School of Medicine at Dartmouth, Lebanon, New Hampshire 03766, USA; 6 Department of Genetics, Institute for Quantitative Biomedical Sciences, Norris Cotton Cancer Center, Geisel School of Medicine at Dartmouth, Lebanon, New Hampshire 03766, USA

**Keywords:** Transcription factor, ChIP-seq, Gene expression

## Abstract

**Background:**

Genetic alterations of transcription factors (TFs) have been implicated in the tumorigenesis of cancers. In many cancers, alteration of TFs results in aberrant activity of them without changing their gene expression level. Gene expression data from microarray or RNA-seq experiments can capture the expression change of genes, however, it is still challenge to reveal the activity change of TFs.

**Results:**

Here we propose a method, called REACTIN (REgulatory ACTivity INference), which integrates TF binding data with gene expression data to identify TFs with significantly differential activity between disease and normal samples. REACTIN successfully detect differential activity of estrogen receptor (ER) between ER+ and ER- samples in 10 breast cancer datasets. When applied to compare tumor and normal breast samples, it reveals TFs that are critical for carcinogenesis of breast cancer. Moreover, Reaction can be utilized to identify transcriptional programs that are predictive to patient survival time of breast cancer patients.

**Conclusions:**

REACTIN provides a useful tool to investigate regulatory programs underlying a biological process providing the related case and control gene expression data. Considering the enormous amount of cancer gene expression data and the increasingly accumulating ChIP-seq data, we expect wide application of REACTIN for revealing the regulatory mechanisms of various diseases.

## Background

Transcription factors (TFs) are a family of proteins that regulate gene expression via binding to specific DNA sequences [1], accounting for 10% of genes in human genome [2,3]. They play instrumental roles in the regulation of many biological processes, such as development, cell proliferation, cell cycle progression, and apoptosis [4-8]. Aberrant expression or activation/inactivation of TFs has been implicated in human diseases [9], particularly, in a variety of different cancer types [10,11]. As a matter of fact, a large number of oncogenes and tumor suppressor genes actually encode TFs. For instance, the most well studied tumor suppressor gene, P53, has been found to be mutated in over 50% of human cancers [12], mostly resulting in impaired capability of transcriptional activation [13].

Microarray experiments have been intensively used to investigate the mechanism of cancer and have resulted in the accumulation of enormous amount of data that are available from the public database such as GEO (Gene Expression Omnibus) [14]. The microarray data enable us to identify differentially expressed genes [15], discover cancer associated gene signatures, and classify tumors into different subtypes [16,17], which have dramatically advanced our understanding about cancer. However, gene expression profiles of tumor samples can only capture the down-stream readout of the driving genetic alterations such as mutations, amplifications and deletions [18]. The changes of the regulatory program underlying a cancer type may not be directly detected from the microarray data. For example, although mutation of P53 gene is known to be a driving event for carcinogenesis in many cancer types [19,20], in most cases there is no significant P53 expression difference between tumor and normal samples: mutation abolishes its transcriptional activity by impacting its DNA binding capacity or protein stability without changing its mRNA expression level [21-23].

Although the alteration of a TF cannot be detected directly from the expression changes of its corresponding mRNA, we expect that alteration of the TF can be revealed by using the expression changes of its target genes. Based on this idea, Rhodes et al. [18] proposed to identify conditional regulatory programs by defining gene expression signatures (differentially expressed genes between cancer and normal samples) and putative TF regulatory signatures (a set of genes containing binding sites of a TF) and examining their overlap. The TFs with enriched regulatory signatures in gene expression signature of cancer were reported to be cancer associated [18]. Previously, we also proposed a Kolmogorov–Smirnov test like method to infer activity changes of TFs based on their binding association with sorted expression profiles [24,25]. All these methods defined TF-gene regulatory relationships by searching putative TF binding sites in promoters of genes, in which the accuracy was affected by both high false positive rate and high false negative rate.

The recent advancement of experimental technologies such as chromatin immunoprecipitation followed by massively parallel DNA sequencing (ChIP-seq) [26] or microarray hybridization (ChIP-chip) [27] enables us to more accurately determine genome wide occupation of TFs. Given the ChIP-seq or ChIP-chip data, we can define a list of target genes for a TF based on the presence of binding peaks in their regulatory DNA regions [28-30]. Alternatively, we can also quantify the binding potential of a TF to all genes, resulting in a continuous representation of TF-gene regulatory relationships [31-33]. In the past few years, a large amount of ChIP-seq and ChIP-chip data for various TFs have been generated by several research consortiums, e.g. ENCODE (The Encyclopedia of DNA Elements) [34,35], and by individual laboratories [36,37].

In this study, we developed a novel method called REACTIN (REgulatory ACTivity INference) to infer TFs that exhibit significantly differential activity in the disease samples versus the normal controls. The REACTIN algorithm integrates case–control gene expression data with TF binding profiles from ChIP-seq or ChIP-chip experiments. To compare the REACTIN algorithm with the other method, we examined the performance of the GSEA method to infer TF activity using the same datasets [38]. We found that GSEA does not always give rise to biological sensible results and the results were often sensitive to the definition of target gene sets of TFs. Further investigation indicated that poor performance of GSEA was mainly caused by the target gene sets of TFs, which were often difficult to be determined based on ChIP-seq or ChIP-chip data. This motivated us to propose the REACTIN algorithm, which applied a similar rank based framework as GSEA but improved it by defining TF-gene regulatory relationship in a quantitative manner.

We evaluated the performance of REACTIN in 10 breast cancer gene expression datasets, all of them containing both ER+ and ER- samples. The method successfully identified ER alpha as the TF that showed significantly higher regulatory activities in ER+ than in ER- breast cancer samples. In addition, it also detected the activity difference of two other breast cancer associated TFs, FOXA1 and GATA3, in ER+ versus ER- samples. Using breast cancer as an example, we also demonstrated that the REACTIN algorithm can be used to investigate regulatory mechanisms governing different breast cancer subtypes by comparison with normal tissues, in addition to identifying TF activity associated with patient survival in their activities. Considering the huge accumulation of microarray data that have been accumulated and the increasingly availability of TF binding data, we expect that REACTIN can be widely used to study the regulatory mechanisms of many human diseases.

## Results

### Overview of REACTIN algorithm for TF activity inference

**Figure 1:**
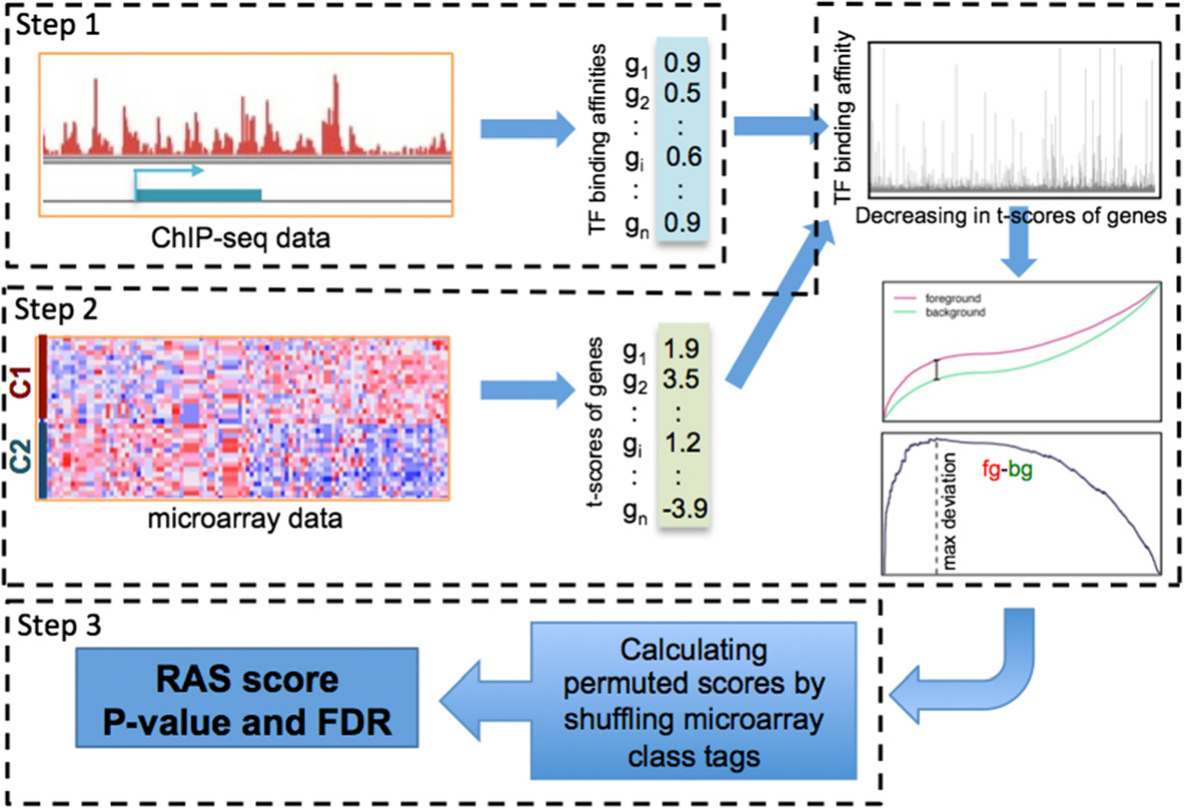
**The workflow of REACTIN algorithm.** Step 1: Measure of the binding affinity of a TF with all human genes using TIP. Step 2: Calculation of regulatory scores (RS) for all TFs. Step 3: Significance estimation and multiple-testing correction.

To investigate the regulatory mechanism underlying a specific cancer type, we developed a method called REACTIN (REgulatory ACTivity INference) to infer TFs that show significantly differential activity in the tumor samples versus the normal controls. Given the case–control gene expression data (cancer versus normal samples), we would expect to see the differential expression of target genes of a TF with altered activity in tumor samples. Similar to the GSEA (Gene Set Enrichment Analysis) method [38], REACTIN ranks all genes based on the expression changes in case versus control samples, and then examines their potential being bound by a TF by referring to its ChIP-seq data. Here the rationale is that it is often difficult and less effective to define a target gene set for a TF due to the quantitative nature of TF-gene interactions [33]. Thus, we used a probabilistic model to predict the target genes of a TF: for each gene we assign a score to measure its probability of being regulated by the TF. Briefly, REACTIN takes a three-step procedure to identify significant genes (Figure 1).

Step 1 The probabilistic measure of TF-gene interactions based on ChIP-seq data. Based on ChIP-seq data, we calculated the binding affinity of a TF with all human genes using a probabilistic model called TIP (Target Identification from Profile) [31]. For a TF, TIP builds a characteristic, average profile of binding around the TSS (Transcription Start Site) of all genes and then uses this profile to weight the sites associated with a given gene, providing a continuous-valued score of this TF for each gene. The score provides a metric that quantifies the binding affinity of a TF with a gene. We have collected 424 human ChIP-seq samples from the ENCODE project [39], based on which we constructed a TF target database that probabilistically measures TF-gene interactions.

Step 2 Calculation of regulatory activity scores (RAS) for all TFs (See Methods). We calculate the t-scores for all genes by comparing their expression levels in case samples and control samples, and sort them to obtain a ranked list. The RAS is calculated by walking down the list and a running-sum statistic is used to capture the correlation between gene expression change (the t-score) and TF binding potential (from ChIP-seq). If genes with higher ranks in the list tend to have higher binding probabilities by a TF, then the TF will achieve a greater RAS score. As a metric, the RAS can be regarded as an extended version of the ES in GSEA analysis.

Step 3 Significance estimation and multiple-testing correction. We estimate the statistical significance of the RAS by using an empirical phenotype-based permutation test procedure that preserves the complex correlation structure of the gene expression data. Specifically, we permute the case/control class labels and recompute the RAS of the gene set in the permuted data, which generates a null distribution for the RAS. The empirical, nominal P value of the observed RAS is then calculated relative to this null distribution. We calculated the false discovery rate by comparing the distribution of the RAS with the null distribution from permutation test.

### Evaluation of REACTIN by comparing ER+ with ER- breast cancer

To test the effectiveness of the REACTIN algorithm, we applied it to identify TFs with significantly different regulatory activities between ER+ and ER- breast cancer samples. Specifically, we collected 10 breast cancer microarray datasets, each containing both ER+ and ER- samples (see Table S1 in Additional file 1: Table S1) [40-49]. ER is a transcription factor that is active in ER+ but not in ER- breast cancer, and its binding profile (ER alpha) is included in our ChIP-seq data collection. If REACTIN is effective, we will identify ER as one of the TFs that shows significantly higher activity in ER+ than in ER- samples.

**Figure 2:**
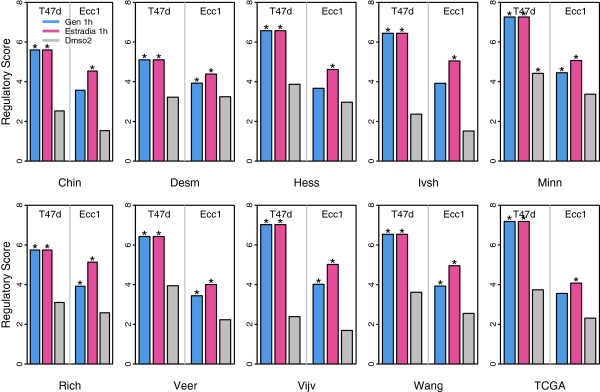
**The activity difference of ER alpha between ER+ and ER- samples in all 10 datasets.** Six ER alpha binding profiles are from T47d or Ecc1 cell lines, and treated with steroid hormone (Gen and Estradia) for 1h or Dmso2 as control. “*” indicates that a ER alpha binding profile has significantly high activity in ER+ than ER- with P<0.01.

Strikingly, REACTIN successfully detected the activity difference of ER alpha between ER+ and ER- breast cancer samples in all of the 10 datasets (Figure 2 and Additional file 2: Table S2). There are 6 ER alpha binding profiles in our ChIP-seq collection, representing binding affinities with human genes in two cell lines (T47d and Ecc1) under three different conditions: two steroid hormone treatments (Gen1h and Estrodia1h) and a control treatment (Dmso2). In all datasets, the ER alpha binding profiles Haib_T47d_Eralphaa_Gen1h and Haib_T47d_Eralphaa_Estradia1h were identified to have significantly higher activity in ER+ than ER- at the 0.25 false discovery rate (FDR<0.25) (Table S2 in Additional file 2: Table S2). For example, Table 1 shows the top 10 most differential TFs between ER+ and ER- in Veer and Wang datasets. As shown, the RAS of Haib_T47d_Eralphaa_Gen1h is 0.624 in the Veer dataset and 6.538 in the Wang dataset, with a FDR of 0.005 and 0.003, respectively. More importantly, there is a clear separation between the significant TF binding profiles and the non-significant ones (Table 1), suggesting a high specificity of the REACTIN algorithm. As shown in the Veer dataset, there are 4 significant TF binding profiles, all of them with a FDR<0.039; but the fifth most differential (based on RAS) profile Haib_T47d_P300_Dmso2 is with a FDR=0.266.

**Table 1:** Top 10 TF binding profiles with significantly different activities between ER+ and ER- breast cancer samples in the Veer and the Wang datasets

**The Veer data**							
**Cell line**	**TF**	**Condition**	**Lab**	**RAS**	**Location**	**P-value**	**FDR**
**T47d**	**Eralphaa**	**Gen1h**	**Haib**	**6.424**	**0.174**	**0**	**0.005**
**T47d**	**Eralphaa**	**Estradia1h**	**Haib**	**6.424**	**0.174**	**0**	**0.011**
**T47d**	**Foxa1**	**Dmso2**	**Haib**	**5.427**	**0.174**	**0**	**0.036**
**T47d**	**Gata3**	**Dmso2**	**Haib**	**5.263**	**0.174**	**0**	**0.039**
T47d	P300	Dmso2	Haib	4.372	0.180	0.002	0.266
Hepg2	Foxa1		Haib	3.905	0.158	0.001	0.413
T47d	Eralphaa	Dmso2	Haib	3.946	0.247	0.012	0.438
Ecc1	Eralphaa	Estradia1h	Haib	4.006	0.126	0	0.457
Mcf7	Cmyc	Estro	Broad	1.710	0.637	0.206	0.695
H1hesc	Ctcf		Broad	1.710	0.106	0.184	0.701
**The Wang data**							
**Cell line**	**TF**	**Condition**	**Lab**	**RAS**	**Location**	**P-value**	**FDR**
**T47d**	**Eralphaa**	**Gen1h**	**Haib**	**6.538**	**0.135**	**0**	**0.003**
**T47d**	**Eralphaa**	**Estradia1h**	**Haib**	**6.538**	**0.135**	**0**	**0.007**
**T47d**	**Gata3**	**Dmso2**	**Haib**	**4.874**	**0.189**	**0.006**	**0.142**
**T47d**	**Foxa1**	**Dmso2**	**Haib**	**5.076**	**0.189**	**0.003**	**0.142**
**Ecc1**	**Eralphaa**	**Estradia1h**	**Haib**	**4.953**	**0.094**	**0**	**0.146**
Ecc1	Eralphaa	Gen1h	Haib	3.929	0.126	0.005	0.872
Hl60	Ctcf		Uw	-1.042	0.704	0.365	0.901
H1hesc	Sin3ak20		Haib	0.735	0.233	0.454	0.921
Gm19239	Ctcf		Broad	-0.917	0.035	0.417	0.801
A549	Gr	Dexd	Haib	0.725	0.281	0.444	0.924

The “Location” column shows the percentile of genes at which the maximum deviation is observed. TF binding profiles with a FDR<0.25 are highlighted in bold.

**Figure 3:**
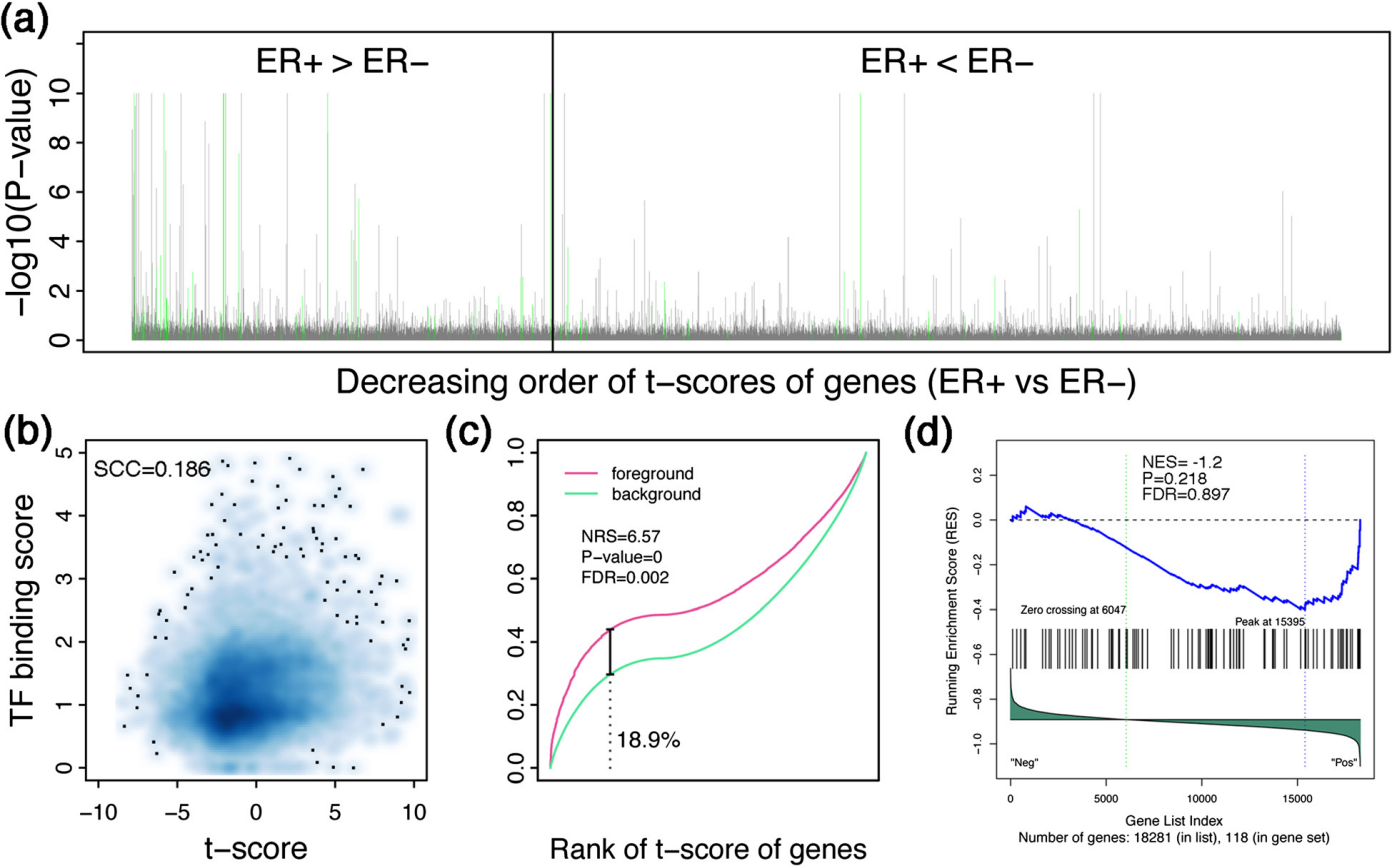
**REACTIN algorithm identifies significant activity difference of ER alpha (Haib_T47d_Eralphaa_Gen1h) in the Hess dataset. (a)** Genes with higher t-scores (ER+ vs ER-) are more likely to be regulated by ER alpha. Genes are sorted in a decreasing order according to their t-scores (ER+ vs ER-). The –log10(P-value) is calculated by TIP, indicating the probability of a gene is bound by ER alpha in Haib_T47d_Eraphaa_Gen1h ChIP-seq data. The green lines indicates ER alpha target genes identified by peak-based method; **(b)** The correlation between the t-scores of genes and TF binding scores calculated by TIP; **(c)** The foreground and background functions for Haib_T47d_Eraphaa_Gen1h binding profile. The foreground and background functions are defined in Formula (xx) and (xx). Note the maximum deviation is obtained at the 18.9% percentile of all genes. **(d)** GSEA results for the ER alpha target gene sets defined by peak-based method (the green lines in **(a)**). Note that it cannot detect the activity difference of ER alpha between ER+ and ER- samples.

In Figure 3, we use Haib_T47d_Eralphaa_Gen1h in the Hess dataset as the example to show how the REACTIN algorithm identifies its activity difference. As shown in Figure 3a, when genes are sorted in the decreasing order of their t-scores (ER+ versus ER- samples in Hess dataset), genes with higher binding possibilities (grey lines with larger –log10 (P-value)) as calculated by TIP [31] are more likely to have higher t-scores (i.e. biased to the left side). This indicates that target genes of ER alpha are more likely to have higher expression levels in ER+ than in ER- samples, implying the regulatory activity difference of ER alpha. In fact, the t-score of genes is significantly correlated with TF binding scores calculated by TIP with a correlation Rho=0.186 (Figure 3b). REACTIN captures such a relationship by comparing a foreground function with a background function as shown in Figure 3c. The maximum deviation of the two functions reflects the activity difference of ER alpha between ER+ and ER- samples (See “Methods” for details).

Target genes determined by peak-based method (the green lines in Figure 3a, genes with a ER alpha binding peaks in [-1000, 500] of their transcriptional start sites) also tend to have higher t-scores. However, GSEA analysis failed to identify ER alpha targets (based on Haib_T47d_Eralphaa_Gen1h binding profile) as a gene set enriched in differentially expressed genes between ER+ and ER- samples in the Hess dataset (Figure 3d). There are two main causes resulting this phenomenon: (1) many of the target genes identified by peak-based method may not be ER alpha targets according to TIP calculated probabilities (Figure 3a); (2) gene set based inference cannot effectively capture the information provided by TF binding data from ChIP-seq experiments.

It is interesting to observe the influence of ChIP-seq cell lines and conditions to the results. The REACTIN algorithm detected more significantly differential activity of ER alpha between ER+ and ER- for binding profiles in T47d than in Ecc1 (Figure 2).

Although both T47d and Ecc1 cell lines maintain estrogen receptors and are steroid-responsive, T47d is derived from breast epithelial tumor cells and Ecc1 is derived from endometrial carcinoma cells. As such, T47d can accurately characterize the ER alpha binding affinities to genes in breast cancer samples. This explains why we observed higher regulatory scores for ER alpha binding profiles from T47d than from Ecc1 in all of the microarray datasets (Figure 2). Additionally, we observed an obvious condition effect: ER alpha activity difference can only be detected when cell lines are treated with steroid hormones during ChIP-seq experiments, because target gene binding of ER alpha can be accurately characterized after treated with hormone than the Dmso2 control (Figure 2).

In addition to ER alpha, we also identified FOXA1 and GATA3 as the TFs with significantly higher activities in ER+ than in ER- breast cancer samples (Table 1 and Additional file 1: Table S1). The association of ER alpha and FOXA1 with breast cancer have been well defined [50-53]. Up to now ER alpha is deemed to be one of the most important molecular factors in the prognosis and therapy of breast cancer [54]. In ER+ breast cancers ER alpha inhibits apoptosis of tumor cells [55]. FOXA1 is a necessary pioneer factor to mediate ER alpha association with compacted DNA in breast cancer cells [56]. When FOXA1 is absent, ER alpha cannot interact with DNA and the expression of ER alpha-mediated genes are prevented [52]. GATA3, has been shown in a recent study to be one of the three genes that are mutated in >10% of breast cancers [49]. A recent study [57] has shown that GATA3 associates directly with ER alpha and progesterone receptor. We compared the mRNA expression levels of ESR1 (ER alpha coding gene), FOXA1 and GATA3 between ER+ and ER- samples in the microarray data, and found that all of them are more highly expressed in ER+ samples. For instance, in the Wang dataset, ESR1, FOXA1 and GATA3 showed significantly higher expression levels in ER+ and ER- samples with P=3e-44, P=4e-16 and P=8e-13, respectively (the Student t-test). These results indicate that the activity difference of these three TFs between ER+ and ER- samples is at least partially caused by the their differential expression at the mRNA level. But we cannot rule out the possibility that in certain samples regulatory activity is a result of point mutation instead of expression change.

### Transcriptional regulatory programs underlying different breast cancer subtypes

We next applied the REACTIN algorithm to investigate the regulatory mechanisms underlying different breast cancer subtypes. We applied it to the microarray data published by Ma et al., which contained expression profiles of 66 samples isolated from ductal carcinoma in situ (DCIS) or invasive ductal carcinoma (IDC) biopsies. The samples can be divided into 6 categories: 14 normal epithelium samples (NE), 9 DCIS-associated malignant epithelium samples (ISE), 9 IDC-associated invasive epithelium samples (INVE), 14 normal stroma samples away from the malignant lesion (NS), 11 DCIS-associated malignant stroma samples (ISS) and 9 IDC-associated invasive stroma samples (INVS).

First, we pooled the samples to obtain 28 normal breast samples (NE and NS) and 38 tumor samples (ISE, INVE, ISS and INVS). We identified TF binding profiles showing significantly differential activities in tumor versus normal breast samples using the REACTIN algorithm. Our results indicate that Stat1 and Stat2 are significantly more active (i.e. their target genes tend to be more highly expressed) in tumor cells than normal cells (Table 2). Members of the Stat family play an important role in regulating breast cancer development [58]. The lack of Stat1 results in aberrant response to IFN signaling and makes the tissue more susceptible to tumor development [59,60]. Stat1 may function as a tumor suppressor via interaction with P53 or BRAC1 [61,62], or by up-regulating P27^Kip1^ expression [63]. In addition, Stat1-deficient mice can spontaneously develop estrogen receptor α-positive luminal mammary carcinomas [59]. At the mRNA level, we found that expression of Stat1 was significantly reduced in tumor cells compared to normal breast tissues (P=2e-8, t-test). Together with the observed activity enhancement of Stat1, we posit that Stat1 might act mainly as a repressor in gene transcriptional regulation.

**Table 2:** TF binding profiles with significantly different activities between breast tumor and normal breast tissues

**Tumor (38) versus normal (28): FDR <0.05**						
**TF**	**Cell line**	**Condition**	**Lab**	**RAS**	**Location**	**P-value**
Stat1	K562	Ifnah6h	Sydh	6.252	0.164	0
Stat2	K562	Ifnah6h	Sydh	6.274	0.164	0
Stat1	K562	Ifnah30	Sydh	5.674	0.159	0
Stat2	K562	Ifnah30	Sydh	5.287	0.157	0
**Tumor stoma (20) versus normal stroma (14): FDR<0.05**						
**TF**	**Cell line**	**Condition**	**Lab**	**RAS**	**Location**	**P-value**
Baf155	Helas3		Sydh	5.095	0.260	0
Gr	A549	Dexc	Haib	5.119	0.225	0.004
Ini1	Helas3		Sydh	4.973	0.252	0.003
Gr	A549	Dexc	Haib	5.125	0.249	0.003
Znf274	Helas3		Sydh	5.207	0.326	0.01
Fosl1	K562		Haib	4.888	0.298	0.003
Pu1	K562		Haib	5.221	0.298	0
**Tumor epithelium (18) versus normal epithelium (14) top 20**						
**TF**	**Cell line**	**Condition**	**Lab**	**RAS**	**Location**	**P-value**
E2f4	Helas3		Sydh	6.151	0.178	0
Stat1	K562	Ifna30	Sydh	5.726	0.235	0
E2f4	K562b		Sydh	5.158	0.165	0
Stat2	K562	Ifna30	Sydh	5.788	0.239	0
Stat1	K562	Ifna6h	Sydh	5.857	0.239	0
Stat2	K562	Ifna6h	Sydh	5.469	0.238	0
E2f1	Helas3		Sydh	5.290	0.167	0
Eralphaa	T47d	Dmso2	Haib	5.042	0.236	0
Irf3	Helas3		Sydh	4.947	0.235	0
E2f1	Mcf7		Sydh	4.962	0.166	0
Stat1	Helas3	Ifng30	Sydh	4.831	0.205	0
Eralphaa	T47d	Gen1h	Haib	4.726	0.167	0.002
Stat1	K562	Ifng6h	Sydh	4.781	0.236	0.002
Nyfb	K562		Sydh	4.751	0.116	0
Baf170	Helas3		Sydh	4.688	0.195	0.004
Eralphaa	T47d	Estradia1h	Haib	4.726	0.167	0.002
Nyfa	K562		Sydh	4.627	0.214	0.
Sta1	K562	Ifng30	Sydh	4.635	0.236	0.001
Sp1	K562		Haib	4.547	0.214	0.001

Next, we compared tumor stroma samples (ISS and INVS) with normal stroma samples (NS). At the significance level FDR<0.05, we identified Baf155, Ini1, Gr, Znf274 and Fosl1. We also compared tumor epithelium samples (ISE and INVE) with normal epithelium samples (NE) using REACTIN. Compared to stroma results, more TFs were identified for epithelium comparison (Table 2). Our results indicate Stat1 and Stat2 show differential activities between tumor and normal breast epithelium samples. E2F1 and E2F4 are also identified as TFs with higher activities in tumor epithelium samples, suggesting higher proliferative rate of malignant epithelium breast cells. E2F1 and E2F4 play the important regulatory role in cell cycle and many studies have demonstrated their importance during tumorigenesis [64,65]. In addition, ER alpha shows higher activity in malignant epithelium cells than in normal epithelium cells. However, no ER alpha activity difference is observed between malignant and normal stroma cells, suggesting that the roles played by ER alpha during breast cancer carcinogenesis is cell type specific.

Finally, we inferred the regulatory activity difference of TFs by comparing ISE with NE, INVE with NE, ISS with NS, and INVS with NS. This analysis gives rise to the regulatory programs underlying each of the breast cancer subtypes (Additional file 3: Table S3).

Our analysis also suggests that the REACTIN algorithm is more sensitive in detecting the differential activity of TFs when applied to more specified cancer subtypes. For example, when the pooled tumor samples were compared against pooled normal samples, only two TFs (Stat1 and Stat2) were identified. However, when INVE samples were compared with NE samples, 192 TF binding profiles showed significant differential activity at the FDR<0.05 significance level (Additional file 3: Table S3). This is due to the heterogeneous nature of tumor samples. Each tumor sample may have its own specific regulatory program, and therefore pooling different tumor samples would reduce the ability of REACTIN to detect the differential regulatory programs (presumably because no single program is shared amongst all these samples). In contrast, tumor samples of the same subtype are more likely to share common regulatory programs and thus be more amenable to detection by the REACTIN algorithm.

### Identification of transcription factors associated with survival of patient with breast cancer

We then adapted the REACTIN algorithm to identify TFs associated with survival of patients with breast cancer. To achieve this, we modified the REACTIN algorithm to calculate a regulatory score of a TF for each samples in a microarray dataset (see “Methods” for details), resulting in an activity profile for each TF binding profile in our ChIP-seq data collection. We calculated the activity scores of all TFs in all samples in the Vijv dataset [46].

**Figure 4:**
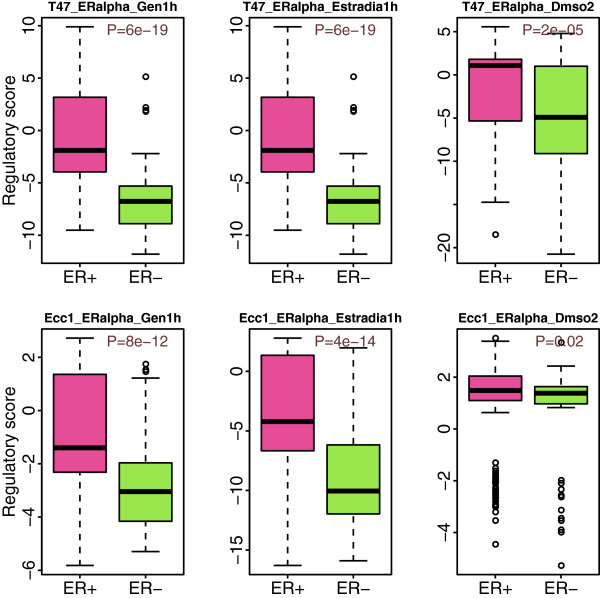
**The activity scores of six TF binding profiles for ER alpha in ER+ and ER-.** The P-values in the top-right corner are calculated based on Wilcox rank sum test. The six TF binding profiles are from two cell lines (T47d and Ecc1) and under three different conditions (treated with steroid hormone Gen/Estradia for 1h, or with Dmso2 as control).

First, we examined whether the inferred activity score of ER alpha reflected its actual activity in a sample. We compared activity scores of ER alpha in ER+ versus ER- breast cancer samples. As shown in Figure 4, the activity scores of ER alpha are significantly higher in ER+ than in ER- samples with the exception of Ecc1_ERapha_Dmso2. This suggests that the inferred activity score of ER alpha can correctly reflect the ER status of a breast cancer sample. We compared the activity scores of all TF binding profiles in ER+ versus ER- samples using Wilcox rank sum test (Additional file 4: Table S4). We find that out of the 424 TF binding profiles we collected, 29 have significantly higher activity scores in ER+ samples and 135 have significantly higher activity scores in ER- samples. Consistent with the results of the REACTIN algorithm, the top three most significant TFs with higher activity scores in ER+ than in ER- are ER alpha, FOXA1 and GATA3.

We then fitted a cox proportional hazard model for each activity profile to identify TFs that were correlated with patient survival time. We identified a total of 35 TF binding profiles that were significantly associated with breast cancer patient survival. Most of them were still significant even after considering the ER status and node status using the multiple variable model (Table 3). Here we use the Sydh_Helas3_E2f4 binding profile as the example to show the relationship between patient survival and E2F4 activity. As shown in Table 3, E2F4 is significantly associated with patient survival with a hazard ratio of 1.291 (95% CI is [1.137, 1.467]) without considering ER status. After considering the ER status and node status, it is still significant with a hazard ratio of 1.237 and a 95% CI of [1.094, 1.4]. In contrast, the expression levels of E2F4 cannot predict patient survival time (P>0.1 for both single and multiple variable Cox regression models).

**Table 3:** TF binding profiles that are significantly correlated with survival of patients with breast cancer (the Vijv dataset)

**Lab_Cell line_TF_Condition**	**P-value**	**Adjusted P value**	**Hazard ratio**	**95% CI**	**P-value**	**Adjusted P value**	**Hazard ratio**	**95% CI**
Sydh_helas3_E2f6	5.96E-06	0.0017	1.073	(1.041, 1.106)	8.44E-05	0.0163	1.064	(1.032, 1.097)
Sydh_K562_Cfos	1.03E-05	0.0017	1.114	(1.062, 1.168)	0.0001	0.0163	1.099	(1.047, 1153)
Sydh_K562_Stat1_Ifng30	1.17E-05	0.0017	1.264	(1.138, 1.403)	0.0002	0.0163	1.221	(1.099, 1.358)
Sydh_K562_Brf2	2046E-05	0.0019	1.172	(1.089, 1.262)	0.0003	0.0163	1.147	(1.065, 1.235)
Sydh_K562_Brf1	2.52E-05	0.0019	1.228	(1.116, 1.350)	0.0003	0.0163	1.192	(1.084, 1.311)
Sydh_K562_Cmyc_Ifng6h	2.62E-05	0.0019	1.322	(1.163, 1.513)	0.0005	0.0168	1.271	(1.111, 1.453)
Sydh_Helas3_Ini1	3.67E-05	0.0020	1.292	(1.144, 1.460)	0.0004	0.0168	1.240	(1.100, 1.396)
Sydh_K562_Tf3c110	3.85E-05	0.0020	1.221	(1.110, 1.343)	0.0003	0.0163	1.193	(1.085, 1.312)
Sydh_K562b_Znf274	4.74E-05	0.0022	1.236	(1.116, 1.369)	0.0006	0.0168	1.198	(1.081, 1.328)
Sydh_K562_Cmyc	5.50E-05	0.0023	1.290	(1.140, 1.461)	0.0006	0.0168	1.242	(1.098, 1.405)
Haib_Gm12878_Atf3	5.84E-05	0.0023	1.230	(1.112, 1.361)	0.0003	0.0163	1.201	(1.088, 1.327)
Sydh_Hepg2_Srebp1	7.36E-05	0.0024	1.211	(1.102, 1.332)	0.0005	0.0168	1.191	(1.080, 1.313)
Sydh_Helas3_E2f4	8.36E-05	0.0024	1.291	(1.137, 1.467)	0.0007	0.0182	1.237	(1.094, 1.400)
Sydh_K562_Cmyc_Ifng30	9.17E-05	0.0024	1.254	(1.120, 1.405)	0.0006	0.0172	1.225	(1.090, 1.376)
Sydh_K562_Cjun_Ifng6h	0.0001	0.0027	1.291	(1.134, 1.469)	0.0014	0.0220	1.233	(1.084, 1.402)
Sydh_K562_Ccnt	0.0001	0.0028	1.345	(1.156, 1.564)	0.0012	0.0220	1.303	(1.110, 1.530)
Sydh_K562b_Gata2	0.0001	0.0028	1.128	(1.061, 1.200)	0.0017	0.0227	1.106	(1.039, 1.178)
Sydh_K562_Irf1_Ifng6h	0.0001	0.0030	1.185	(1.086, 1.293)	0.0009	0.0215	1.159	(1.062, 1.624)
Sydh_K562_Bdp1	0.0002	0.0033	1.233	(1.106, 1.375)	0.0020	0.0246	1.190	(1.066, 1.330)
Sydh_K562_Rpc155	0.0002	0.0035	1.410	(1.177, 1.689)	0.0012	0.0220	1.353	(1.127, 1.624)
Haib_Hepg2_Rxra	0.0002	0.0035	1.210	(1.095, 1.338)	0.0013	0.0220	1.179	(1.067, 1.303)
Sydh_K562_Hmgn3	0.0002	0.0035	1.180	(1.082, 1.288)	0.0156	0.0620	1.122	(1.022, 1.233)
Uw_K562_Ctcf	0.0002	0.0035	1.298	(1.131, 1.490)	0.0013	0.0220	1.268	(1.097, 1.465)
Haib_Hepg2_Fosl2	0.0002	0.0039	1.419	(1.177, 1.711)	0.0015	0.0220	1.360	(1.125, 10645)
Haib_Hepg2_Hdac2	0.0003	0.0040	1.178	(1.079, 1.286)	0.0019	0.0241	1.150	(1.053, 1.255)
Sydh_K562_Brg1	0.0003	0.0040	1.106	(1.048, 1.168)	0.0038	0.0317	1.085	(1.027, 1.358)
Haib_Hepg2_Gabp	0.0003	0.0043	1.238	(1.103, 1.390)	0.0017	0.0227	1.207	(1.074, 1.358)
Sydh_Gm12878_Ctcf	0.0003	0.0045	1.333	(1.140, 1.558)	0.0012	0.0220	1.290	(1.106, 1.504)
Haib_Hepg2_Sp1	0.0003	0.0045	1.153	(1.067, 1.247)	0.0014	0.0220	1.135	(1.050, 1.226)
Haib_Gm12878_Bcl11a	0.0003	0.0045	1.220	(1.094, 1.361)	0.0012	0.0220	1.194	(1.072, 1.329)
Sydh_Mcf7_Hae2f1	0.0004	0.0045	1.224	(1.095, 1.367)	0.0004	0.0168	1.228	(1.096, 1.376)
Sydh_Helas3_Cjun	0.0004	0.0045	1.075	(1.033, 1.118)	0.0028	0.0293	1.063	(1.021, 1.106)
Haib_Hepg2_Sin3ak20	0.0004	0.0045	1.368	(1.151, 1.626)	0.0014	0.0220	1.323	(1.114, 1.571)
Sydh_K562_Stat1_Infa6h	0.0004	0.0045	1.273	(1.114, 1.455)	0.0032	0.0295	1.222	(1.069, 1.397)
Sydh_Ntd1_Znf274	0.0004	0.0049	1.204	(1.086, 1.336)	0.0026	0.0286	1.174	(1.058, 1.303)

The hazard ratio and 95% confidence interval (CI) are calculated using cox proportional hazards model with single variable or multiple variables. In the multiple cox regression model, ESR (Estrogen receptor status) and Posnodes (lymph node status) are included as variables together with the inferred TF activity scores.

**Figure 5:**
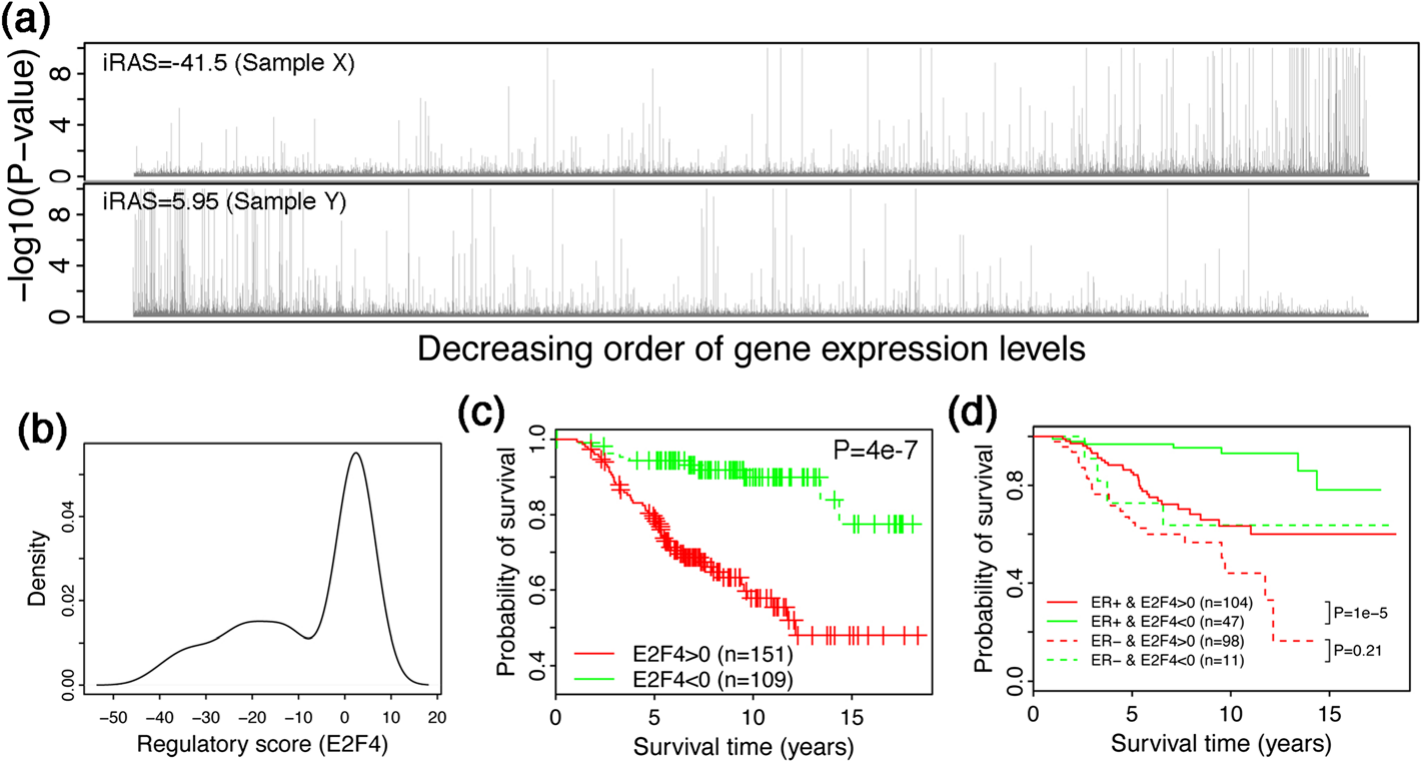
**The relationship between survival time of patients with breast cancer and inferred E2F4 activity score. **The Vijv dataset is used in the calculation. **(a)** Two breast cancer samples with an E2F4 regulatory score of -41.5 and 5.95, respectively. **(b)** Distribution of E2F4 activity scores in the 260 samples. **(c)** The survival curves of patients with breast cancer. “E2F4>0” shows patients with positive E2F4 activity scores; “E2F4<0” shows patients with negative E2F4 activity scores. **(d)** The survival curves of four categories patients: ER+ & E2F4>0, ER+ & E2F4<0, ER- & E2F4>0 and ER- & E2F4<0. E2F4 activity score is inferred based on the Sydh_Helas3_E2F4 binding profile.

Out of the 260 breast cancer samples of the Vijv dataset, 151 have a positive E2F4 activity score and 101 have a negative E2F4 activity score. Figure 5a shows two samples with an E2F4 regulatory score of -41.5 and 5.95, respectively. As shown, in the sample with a negative score genes with higher probability of being E2F4 regulated are more likely to have lower expression levels (i.e. biased to the right side), whereas in the sample with a positive score the opposite is observed. Distribution of E2F4 activity scores in the 260 samples is shown in Figure 5b. Patients with positive E2F4 activity scores (E2F4>0) demonstrate significantly shorter survival time than patients with negative E2F4 activity scores (E2F4<0) with a P-value of 4e-7 (Figure 5c). We note that the ER+ patients show significant longer survival time than ER- patients with P-value of 4e-6. In another words, the inferred activity score of E2F4 achieves better accuracy than ER status when predicting survival time of patients. Combining E2F4 activity score with ER status, we divided samples into four categories as shown in Figure 5d. In ER+ samples, patients with positive E2F4 activity still have significantly shorter survival time than those with negative E2F4 activity (P=1e-5). In ER- samples, the same trend can also be observed, although the difference is not significant due to small sample size.

### Comparison with other methods

The REACTIN algorithm extends GSEA analysis by adopting a probabilistic representation of gene sets. Namely, instead of using a defined gene set, it regards all genes as members of a gene set with different probabilities. This extension is very suitable for the TF-gene regulatory relationships defined by ChIP-seq or ChIP-chip experiments. In fact, there exist both strong and weak binding of TFs with their targets. As such TF-gene interactions should be represented in a quantitative rather than in a binary manner. Despite of this, we defined a target gene set for all TF binding profiles in our ChIP-seq collection for comparison purpose. For a TF, target genes are defined as those with at least one binding peak of the TF in its promoter regions (from 1000 bp upstream to 500 bp downstream of their TSS). We then applied GSEA analysis to identify TFs with target gene sets enriched in differential expression profiles between ER+ and ER- breast cancers. It is observed that GSEA is less sensitive: it identified ER alpha as the significant TFs in only 7 of the 10 datasets. It failed to identify ER alpha in the Desm, Hess and Ivsh datasets (Additional file 5: Table S5). It also failed to identify FOXA1 and GATA3 in all of the 10 datasets.

We also performed GSEA analysis using TF target gene sets defined by TIP method, which failed to identify ER alpha in 5 of the 10 datasets (Additional file 6: Table S6). In addition, we examined the TF target gene sets defined by the ChEA database. GSEA analysis using these gene sets identified ER alpha in only one dataset (Additional file 7: Table S7). Thus, we conclude that the REACTIN algorithm outperforms GSEA analysis in identifying regulatory programs underlying cancer.

## Discussion

In this article, we have introduced a new algorithm named REACTIN that integrated gene expression data with TF binding data to identify TFs with significantly differential activity between two sample groups. REACTIN operates under a similar framework to that of GSEA, which has been widely used to identify gene sets associated with an interested phenotype. Theoretically, we can utilize GSEA analysis to identify disease associated TFs, if we define a gene set for each TF. However, we instead adopt a quantitative method to measure TF-gene regulatory relationships in REACTIN for two reasons. First, as with many biological processes, TF-gene binding events should be regarded as a quantitative and continuous variable rather than a simple on/off switch model. For any given TF, there exists strong and weak binding sites in the genome, both contributing to transcriptional regulation of genes. Thus, we believe a continuous representation of TF-gene binding can be more accurately reflect the overall nature of TF-gene regulatory relationships. Second, it is often technically difficult to set up a cut-off for defining target genes of a TF based on its ChIP-seq data. For example, given the ChIP-seq data of a TF, we usually define target genes as those with one or more binding peaks in their promoter regions. The definition of promoter region is often arbitrary, e.g. 1 kb or 5 kb around the TSS have been used in previous publications. For these two reasons, the gene set based method cannot fully utilize the TF binding information derived from ChIP-seq data and is ultimately less robust than REACTIN, as has been shown by our results (Additional file 5: Table S5 and Additional file 7: Table S7).

The REACTIN algorithm calculates a regulatory score and assesses its significance for each TF by comparing and permuting the case and control samples. In this article, we also showed a modified version of this algorithm. In the modified version, we calculated a regulatory score of a TF for each individual sample, yielding an activity profile of the TF. Given the profile, we can compare its inferred activities in case and control samples (e.g. using Wilcox rank sum test) to examine the activity difference of a TF (Additional file 4: Table S4). Then, what is the relationship of the two methods for identifying TFs with significant differential activities between case and control? This is analogous to the problem of identifying differentially expressed genes from case–control microarray data. In general, two categories of methods have been widely used. One is to directly compare expression levels of genes between case and control using t-test or Wilcox rank sum test. The other category, typified by SAM (Statistical Analysis of Microarrays) [66], adopts a permutation-based strategy to take into account correlation structure and correct for multiple testing. The REACTIN algorithm applies a similar strategy as SAM, while the modified method is similar to the test based method for identifying differential expressed genes. Practically, the results REACTIN is much more conservative than the modified method, resulting in only the most confident TFs. The modified version identifies more TFs that show differential activities between case and control (Additional file 3: Table S3). In addition, it is more flexible by providing TF activity information in each individual sample. For example, as we have shown, it can be used to identify TFs associated with patient survival (Additional file 8: Table S8).

In Figure 2, we show that the cell line and the conditions governing ChIP-seq experiments would result in different TF binding profiles and impact the REACTIN results. In fact, bindings of many TFs are tissue specific and condition dependent. For example, a TF may have different targets genes in tumor and normal tissues. Ideally, we should use ChIP-seq data from tissue and conditions that match with the gene expression data. For example, as shown ChIP-seq in T47d (steroid hormone treated) can more accurately capture TF-gene binding relations in breast cancer than ChIP-seq in Ecc1 cell lines.

## Conclusion

In this study, we proposed a method called REACTIN to identify TFs that exhibit significantly differential activity between the disease samples versus the normal controls. The method integrates the case-control gene expression data with the TF binding data from ChIP-seq experiments. We applied REACTIN to compare ER+ with ER- samples in ten breast cancer datasets and successful detected the activity difference of ER alpha, FOXA1 and GATA3 between ER+ and ER- breast cancer subtypes. We also demonstrated the effectiveness of applying REACTIN to identify TFs that were predictive to survival times of patients with breast cancer. REACTIN provides a useful tool to investigate regulatory programs underlying a disease, and more generally underlying any biological processes, providing the related case and control gene expression data. As the available pool of ChIP-seq data increases, we expect a wide application of the REACTIN algorithm to the study of regulatory mechanisms governing different human diseases.

## Methods

### Microarray gene expression datasets

All the microarray data used in this study were downloaded from the public Gene Expression Omnibus (GEO) databases or from the websites provided by the original publications (Additional file 1: Table S1). In most of the datasets gene expression was measured as absolute values by one-channel arrays, while in the others relative expression levels were provided from two-channel cDNA arrays. The two types of datasets were processed in different ways. In microarray data, some genes are represented by multiple probesets. When multiple probe sets were mapped to the same gene, their values were averaged to represent the expression level of the gene for datasets from two-channel arrays, whereas the probe set with the maximum average value was used for datasets from one-channel arrays.

### Calculation of binding affinity of transcription factors with genes

ChIP-seq data provide binding information of a TF at each nucleotide in a genome (i.e. the number of reads covering the nucleotide). Based on ChIP-seq data, we calculated the binding affinity of a TF with all human Refseq genes using a probabilistic model called TIP (Target Identification from Profile) [31]. For a TF, TIP builds a characteristic, averaged profile of binding around the TSS of all genes and then uses this profile to weight the sites associated with a given gene, providing a continuous-valued score of this TF for each gene. The score provides a metric that quantifies the binding affinity of a TF with a gene. We downloaded a total of 424 ChIP-seq track files from the ENCODE project [39,67], representing binding profiles of >120 human TFs in different cell lines or conditions. For each track file, we applied TIP [31] to calculate the binding affinities of the TF with all human Refseq genes, resulting in a total of 424 TF binding affinity profiles. We note that the binding profile of a TF can also be calculated based on the ChIP-chip data of the TF in the same way.

### Transformation of binding affinity to binding score

We represented the above calculated binding affinity profiles as a matrix |A|, where a_i,j_ was the binding affinity of TF j with gene i. Then we calculated the probability of a gene being regulated by a TF. Given the binding affinity profile of the j_th_ TF, a_•,j_, we calculated the z-score for gene i as z_i,j_ = (a_i,j_-mean(a_•,j_))/sd(a_•,j_), where mean(a_•,j_) and sd(a_•,j_) are respectively mean and standard deviation; and then estimated the corresponding p-value by referring to a standard normal distribution.

This resulted in a p-value matrix |P| containing the p-values for all genes in all ChIP-seq tracks. The elements in |P| was subsequently transformed into non-negative values by taking –log10 (P_i,j_) and truncated at 10 (namely, set the value to 10 if –log10(P_i,j_)>10) to obtain the matrix |S′|. The goal of truncation at 10 is to avoid the influence of extreme values in subsequent analysis. After this transformation, -log10 (P) follows an exponential distribution.

Finally, we normalized |S′| into a matrix |S| by the following s_i,j_ = (s'_i,j_-min(S'))/(max(S')-min(S')), where min(S') and max(S') are minimum and maximum values of |S'|, respectively. After this transformation, all elements in |S| take a value raging from 0 to 1, with a higher value of s_i,j_ indicating a higher probability of gene i being regulated by the corresponding TF in ChIP-seq track j.

### Regulatory activity inference (REACTIN) for transcription factors

Many gene expression experiments follow a case–control design to investigate the mechanism of a disease, where expressions of genes are compared between a case group (e.g. tumor samples from breast cancer patients) and a control group (e.g. samples from normal breast tissues). With this type of data, we propose a method called REACTIN (REgulatory ACTivity INference) to identify transcription factors (TFs) that show significantly differential activities between the case and the control groups. REACTIN calculates a RAS for a TF using a procedure similar to the gene set enrichment analysis (GSEA) [38]. In contrast to the GSEA method, REACTIN does not take a gene set (e.g. the target gene list of a TF) as the input, instead, it utilizes the regulatory potential of a TF to all genes calculated from ChIP-seq data. In practice, the |S| matrix described in the preceding section is used.

Given a gene expression data (a case–control data with n_1_ case and n_2_ control samples) and the regulatory potential profile of a TF (a column from matrix |S|, denoted as s=(s_1_, s_2_, …, s_g_), where g is the total number of genes), we calculate the RAS for this TF using the following procedure.

1) We calculated the t-score for each gene by comparing its expression levels in the two sample groups, $t=\frac{\mu_{1}-\mu_{2}}{\sqrt{\delta_{1}^{2}/n_{1}+\delta_{2}^{2}/n_{2}}}$, where μ_1_, μ_2_ are means and δ_1_, δ_2_ are standard deviations of expression levels of the gene in case and control samples. Subsequently, all genes are sorted in the decreasing order of their t-scores to obtain a ranked t-score vector t'=(t_(1)_, t_(2)_, …., t_(g)_), and accordingly, the vector s is reordered to s'=(s'_1_, s'_2_, …, s'_g_). In some case control data, gene expression is measured in paired samples (e.g. tumor versus adjacent normal tissue). In these cases, we used paired t-test to calculate the t-scores of genes to increase the statistical power: $t=\frac{\mu_{d}}{\delta_{d}}$, where d_i_ is the expression difference of a gene between the matched case and control of sample i.

2) We defined two non-decreasing functions, a foreground function f(i) and a background function b(i), based on the two vectors t' and s' as follows:

3)

$$f\left(i\right)=\frac{\sum_{k=1}^{i}\left|t_{\left(k\right)}2s{'}_{k}\right|}{\sum_{k=1}^{g}\left|t_{\left(k\right)}2s{'}_{k}\right|},1\leq i\leq g;$$

4)

$$b\left(i\right)=\frac{\sum_{k=1}^{i}\left|t_{\left(k\right)}2\left(1-s^{'}{}_{k}\right)\right|}{\sum_{k=1}^{g}\left|t_{\left(k\right)}2\left(1-s^{'}{}_{k}\right)\right|},1\leq i\leq g.$$

5) We compared the difference between the foreground function and the background function at each position i to obtain the maximum value (ps^+^) and the minimum value (ps^-^) of f(i)-b(i).

6) *ps*^+^ = *max*(*f*(*i*_*max*_) - *b*(*i*_*max*_), 0), where $i_{\text{max}}=\text{arg}\underset{i=1,2,\ldots ,g}{\text{max}}\left\{f\left(i\right)-b\left(i\right)\right\}$.

7) *ps*^+^ = *max*(*f*(*i*_*max*_) - *b*(*i*_*max*_), 0) where $i_{\text{min}}=\text{arg}\underset{i=1,2,\ldots ,g}{\text{min}}\left\{f\left(i\right)-b\left(i\right)\right\}$.

8) A pre-score is then defined as ps=ps^+^, if ps^+^>|ps^-^|; and otherwise ps=ps^-^.

9) Normalization of pre-score into a normalized score, which we denoted as regulatory activity score (RAS). To achieve this, we shuffled the sample tags of the two groups, re-calculated the t-scores for all genes and repeated step 1–3 to obtain a permuted ps^+^ and a permuted ps^-^. Specifically, we used a balanced permutation strategy by randomly selecting n_1_/2 samples from case group and n_2_/2 samples from the control groups to form the shuffled case group and using the remaining samples as the shuffled control group. We performed a total of m times of permutations (e.g. m=1000), resulting in a vector ps^perm+^ and a vector ps^perm-^. The RAS was calculated by normalizing against the permuted ps-scores:

10) *RAS* = *ps*/*mean*(*ps*^*perm* +^), if ps≥0; and otherwise, *RAS* = *ps*/|*mean*(*ps*^*perm* -^)|.

11) Calculation of p-value for RAS. The significance of a RAS can be calculated based on the m times of permutations. If ps≥0, then p-value is calculated as the fraction of elements in ps^perm+^ that are equal to or greater than ps; otherwise, p-value is calculated as the fraction of elements in ps^perm-^ that are equal to or less than ps.

12) Calculation of false discovery rate (FDR). Typically, we would calculate the RAS for a large number of TFs (e.g. in matrix |S|, we have 424 TF binding profiles generated by the ENCODE project). To correct for multiple testing, we calculated the FDR of each TF, also based on permutations. The RAS for a TF is a normalized score and is directly comparable between different TFs. Thus, we normalized the permutated pre-scores in ps^perm+^ and ps^perm-^ for all TFs and used them to calculate the FDR of each TF. For a TF with regulatory activity score of RAS*≥0, its FDR was calculated as the ratio of the percentage of all normalized ps^perm+^ scores with a value ≥RAS*, divided by the percentage of observed RASs with a value ≥RAS*; and similarly for a TF with RAS*<0.

### Calculation of individual regulatory activity score of transcription factors in each sample

In some cases, we need to calculate the regulatory activity scores of a TF in each of the samples. For example, in order to identify TFs associated with patient survival, we may calculate the individual RASs (we called them iRASs to discriminate them from the iRASs calculated by case versus control comparison) of TFs in all samples and then correlate them with patient survival times. To achieve this purpose, we modified the above-described procedure to calculate sample-wise iRASs for TFs.

For microarray data that measures the relative expression levels of genes (e.g. data from experiments using two channel cDNA arrays), we just replaced the t-score profile with each individual relative expression profile in step 1 to calculate the pre-scores of TFs in that sample (step 2–3). To normalize the pre-score, we permuted genes to obtain shuffled gene expression profiles, calculated the permuted pre-scores and normalized the observed scores based on them using a similar method as described in step 4.

If the microarray data provide the absolute values of gene expression levels (e.g. data from one-channel Affymetrix arrays), we performed an additional normalization step to transform absolute expression into relative expression levels. Specifically, we first performed quantile normalization [68] for all expression profiles, so that each sample in the dataset achieved exactly the same distribution. Then for each gene, we divided its values by the median expression levels in all samples and took log10 transformation to obtain the relative expression levels. Subsequently, the iRASs of TFs in all samples can be calculated using the method described in the previous paragraph.

### Identification of transcription factors associated with patient survival

Given the calculated iRAS profiles of TFs in all samples, we used the Cox proportional hazards model to identify TFs that were predictive to survival time of patient with breast cancer. The model was designed in the following form: $\text{log}h\left(t\right)=\text{log}h_{0}\left(t\right)+\beta^{'}x^{'}+\overset{p}{\underset{i=1}{\sum }}\beta_{i}x_{i}$, where x' is the RAS profile for a TF, x=(x_1_, x_2_, …, x_p_) are p confounding factors (e.g. tumor size, estrogen receptor status, etc.), h_0_(t) represents basic function of risk, and h(t) represents hazard rate at time t. We examined the iRAS profiles of all TFs one by one, each time including a single TF and a selected list of confounding factors as the predictive variables. The TFs with significant coefficient (P<0.001) were identified as those associated with patient survival. These TFs provide additional predictive power than the confounding factors. In practice, we only selected the significant clinical features as the confounding factors in our model. The significant clinical features are those with P<0.01 in a full model that includes all the confounding features. In the Vijv dataset, ER status and positive node status are the two clinical features that are significant and included as the confounding factors. The R package “survival” was used to implement the Cox regression model.

The algorithm in this paper is coded in C++ and is available from http://www.dartmouth.edu/~chaocheng/software/reactin/reactin.html.

## Abbreviations

ChIP-chip: Chromatin immunoprecipitation followed by massively parallel microarray hybridization; ChIP-seq: Chromatin immunoprecipitation followed by massively parallel DNA sequencing; ENCODE: The Encyclopedia of DNA Elements; ER: Estrogen receptor; FDR: False discovery rate; GSEA: Gene set enrichment analysis; DCIS: Ductal carcinoma in situ; IDC: Invasive ductal carcinoma; INVE: IDC-associated invasive epithelium; INVS: IDC-associated invasive stroma; ISE: DCIS-associated malignant epithelium; ISS: DCIS-associated malignant stroma samples; NE: Normal epithelium samples; NS: Malignant lesion; RAS: Regulatory activity score; REACTIN: Regulatory activity inference; SAM: Statistical analysis of microarrays; TF: Transcription factor; TIP: Target identification from profile; TSS: Transcription start site.

## Competing interests

The authors declare that they have no competing interests.

## Authors’ contributions

CC conceived and designed the study. CC and MZ performed the full analysis. CC, MZ and CL wrote the manuscript. All authors read and approved the final manuscript.

## Supplementary Material

Additional file 1: Table S1 Breast cancer datasets used in this study. Click here for file

Additional file 2: Table S2 Activity differences of TF profiles between ER+ and ER- breast cancer samples in 10 datasets. In each dataset, the top 10 TF profiles are shown. Significant profiles (FDR<0.25) are highlighted in blue color. Click here for file

Additional file 3: Table S3 Activity differences of TF profiles between different breast cancer subtypes and normal breast tissue in Ma dataset. The data contains 6 pairs of comparison: ISE vs. NE, INVE vs. NE, ISE+INVE vs. NE, ISS vs. NS, INVS vs. NSS, and ISS+INVS vs. NS. NE: normal epithelium; NS: normal stroma; ISE: in situ epithelium; INVE: invasive epithelium; ISE: in situ stroma; INVS: invasive stroma. Click here for file

Additional file 4: Table S4 Difference of activity scores of TF binding profiles between ER+ and ER- samples in Vijv dataset. Given a TF binding profile, a regulatory score is calculated for each sample and then the scores are compared between ER+ and ER- samples using the one-sided Wilcox rank sum test. Click here for file

Additional file 5: Table S5 GSEA results using TF target gene sets defined based on peak-based method. For each TF binding profile, target genes are defined as those with at least one binding peaks in their promoter regions (1kb upstream to 500bp downstream of TSS of genes). Peakseq method is used to determine binding peaks. ES: enrichment score; NES: normalized enrichment score. Click here for file

Additional file 6: Table S6 GSEA results using TF target gene sets identified by TIP algorithm. Click here for file

Additional file 7: Table S7 GSEA results using TF target gene sets provided from ChEA database. Click here for file

Additional file 8: Table S8 Association of TF activity scores with survival time of patient with breast cancer in Vijv dataset. Given a TF binding profile, a regulatory score is calculated for each sample. The association of a TF profile with patient survival is calculated using cox proportional hazard regression model. In the simple cox regression model, the TF activity score is used as the single variable. In the multiple cox regression model, TF activity score, ER status (ER+ or ER-) and lymph node status (+ or -) are used as variables. The hazard ratio and its 95% confidence interval are provided. P-values are adjusted using Benjamini Hochberg multiple testing correction. Click here for file
